# Audiovisual integration of speech: evidence for increased accuracy in “talk” versus “listen” condition

**DOI:** 10.1007/s00221-025-07088-7

**Published:** 2025-05-26

**Authors:** Lefteris Themelis Zografos, Anna Konstantoulaki, Christoph Klein, Argiro Vatakis, Nikolaos Smyrnis

**Affiliations:** 1 Laboratory of Cognitive Neuroscience and Sensorimotor Control, University Mental Health Neurosciences and Precision Medicine Research Institute “COSTAS STEFANIS”, Athens, Greece; 2 Multisensory and Temporal Processing Laboratory (MultiTimeLab), Department of Psychology, Panteion University of Social and Political Sciences, Athens, Greece; 3 2nd Psychiatry Department, National, and Kapodistrian University of Athens, Medical School, University General Hospital «AΤΤΙΚΟΝ», Athens, Greece; 4 Department of Child and Adolescent Psychiatry, University of Freiburg, Freiburg im Breisgau, Germany; 5 Department of Child and Adolescent Psychiatry, Medical Faculty, University of Cologne, Cologne, Germany

**Keywords:** Multisensory integration, Temporal binding window, Self-generated action, Movement, Speech

## Abstract

Processing of sensory stimuli generated by our own actions differs from that of externally generated stimuli. However, most evidence regarding this phenomenon concerns the processing of unisensory stimuli. A few studies have explored the effect of self-generated actions on multisensory stimuli and how it affects the integration of these stimuli. Most of them used abstract stimuli (e.g., flashes, beeps) rather than more natural ones such as sensations that are commonly correlated with actions that we perform in our everyday lives such as speech. In the current study, we explored the effect of self-generated action on the process of multisensory integration (MSI) during speech. We used a novel paradigm where participants were either listening to the echo of their own speech, while watching a video of themselves producing the same speech (“talk”, active condition), or they listened to their previously recorded speech and watched the prerecorded video of themselves producing the same speech (“listen”, passive condition). In both conditions, different stimulus onset asynchronies were introduced between the auditory and visual streams and participants were asked to perform simultaneity judgments. Using these judgments, we determined temporal binding windows (TBW) of integration for each participant and condition. We found that the TBW was significantly smaller in the active as compared to the passive condition indicating more accurate MSI. These results support the conclusion that sensory perception is modulated by self-generated action at the multisensory in addition to the unisensory level.

**Supplementary Information:**

The online version contains supplementary material available at 10.1007/s00221-025-07088-7.

## Introduction

Multisensory integration (ΜSΙ) refers to the process by which sensory inputs from different modalities are combined to construct a unified sensory experience that emerges from the interaction of these inputs and cannot be “deconstructed” to reconstruct the component inputs from which it was created (Stein and Meredith 1993; Stein et al. 2009). The operational definition of MSI that can be applied both at the behavioral and the neural level is a significant difference in the response evoked by the cross-modal combination of sensory stimuli compared to the response evoked by the most effective of its components individually (Stein et al. 2009, 2014). Cross-modal stimuli that are proximate in space and time, are most likely to be derived from the same external event and their unification into a single percept by MSI improves the ability of the organism to detect and respond to this external event (Stein et al. 2014).

Several behavioral paradigms have been developed to study MSI in humans. Many paradigms study the difference in response time between unimodal and bimodal stimuli presentation as an indication of MSI and have shown that faster reaction times tend to accompany the presentation of bimodal stimuli (Canzoneri et al, 2012; Galigani et al. 2020; Noel et al. 2022). Other paradigms use time proximal cross modal sensory stimuli to study MSI induced sensory fusion effects. In the Mc-Gurk illusion (McGurk & McDonald, 1976) the combination of an auditory syllable presentation (/ba/) with the simultaneous visual presentation of a person uttering of another syllable (/ga/) leads to the illusory audiovisual percept of a third syllable (/da/, /ta/, or /tha/). In the double flash illusion (or sound-induced flash illusion; SIFI) the synchronous presentation of a single visual flash with multiple auditory beeps evokes the illusory percept of multiple flashes (“fission” illusion), while the synchronous presentation of two flashes and one beep evokes the perception of a single flash being presented (the so-called “fusion” illusion), (Shams et al. 2000).

In the simultaneity judgment (SJ) task used in the current study, observers assess whether auditory and visual stimuli, presented with different time intervals between them (i.e. stimulus onset asynchronies, SOAs), are perceived as simultaneous (e.g. Kostaki and Vatakis 2018; Wallace and Stevenson 2014). The task essentially establishes a window of time named temporal binding window (TBW) during which multisensory stimuli are likely to be perceived as bound together or integrated (Conrey and Pisoni, 2006; Diederich et al. 2009). This task offers the advantage compared to the Mc-Gurk illusion and the double flush illusion of establishing the degree of MSI integration of the cross-modal stimuli into a single percept, which is determined by the width of the TBW (Horsfall et al. 2021). The TBW width is generally influenced by the type of stimuli presented (e.g. Vatakis et al. 2012; Vatakis and Spence 2006). In general, a narrower TBW indicates higher accuracy in MSI integration allowing for the integration into a single percept of truly synchronous stimuli (Zhu et al. 2023; Powers et al. 2009). Narrower TBW are usually observed for simple stimuli such as flashes and beeps, whereas wider windows are noticed with more complex stimuli such as speech (Vatakis and Spence 2006, 2008;2010; Stevenson and Wallace 2013; De Niear et al. 2018). In addition, perceptual learning has been shown to narrow the TBW of integration (McGovern et al. 2016). Narrower auditory TBW were also observed in musicians compared to non-musicians and were interpreted as evidence of increased MSI accuracy for musicians due to their training (Lee and Noppeney 2014).

**Fig. 1:**
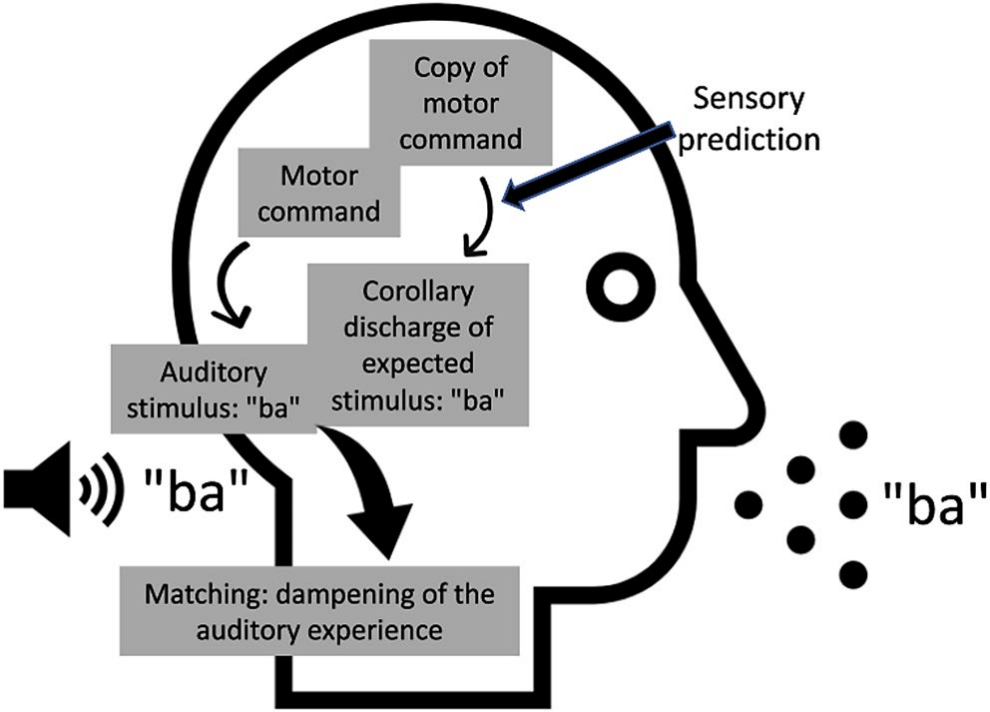
Representation of the corollary discharge mechanism during speech. An efferent copy of the planned speech sound is sent from Broca’s area to the auditory cortex where it results in corollary discharge. The actual movement - related sound (i.e. speech, depicted on the back of the head) arrives simultaneously or few milliseconds after the corollary discharge to the auditory cortex. If the corollary discharge matches the auditory reafferent, the sensory experience is suppressed

One critical aspect of sensory processing of both unisensory and multisensory stimuli is the separation of sensory information that relates to our own actions from sensory information that originates from the external environment. In simple terms, for every sensory input that we are receiving, we need to distinguish if it was caused by our actions or not. Previous studies have shown that sensory processing of self-generated stimuli differs from that of externally generated stimuli (Blakemore et al. 1998a, b, 1999; Wolpert and Flanagan 2001). Thus, we can predict the sensory output from our own movements and prepare for future actions (Wurtz and Sommer 2004). The neurophysiological substrate of this prediction is believed to originate in the so called “corollary discharge”, which is an efference copy of the motor command that is transmitted to the sensory processing areas (Parlikar et al. 2019; Wurtz and Sommer 2004). For instance, when we talk, our motor system sends an efference copy of the speech related motor command to the auditory sensory areas. This copy generates a corollary discharge of the expected auditory sensation that is responsible for a dampening (suppression) of the auditory sensation generated by our own voice (see Fig. 1; Ford and Mathalon 2005, 2019; Jack et al. 2019; Scott 2013). The same phenomenon is also observed in the somatosensory system where self-generated tactile stimuli are perceived as weaker relative to the same intensity tactile stimuli generated externally (Blakemore and Wolpert 1998b, 2000).

While the effect of self-generated action on unisensory stimuli, namely suppression of the perceived intensity, is well-established, the effect of self-generated action on multisensory integration is relatively unexplored. The question arises whether self-generated multisensory stimuli produce the same MSI effects comparted to externally generated stimuli. One could hypothesize that expectation and prediction of the sensory effects for self-generated cross modal stimuli would result in the modulation of MSI being more accurate compared to MSI for externally generated cross modal stimuli.

Two studies (van Kemenade et al. 2016; Straube et al. 2017) examined the effect of self-generated action on the prediction of unisensory and multisensory stimuli. Participants pressed a button (self-generated action) after which they were presented with unisensory (visual or auditory) or multisensory (audiovisual) stimuli either synchronously or with varying delays and they had to report the detection of a delay between their action and the sensory stimuli. In a second experiment, participants could either press the button actively or passively (a device was pushing the participants’ finger). The authors observed that predictions of delay were more accurate for multisensory compared to unisensory stimuli and for active compared to passive button presses. The authors concluded that a forward model of the self-generated action, related to the efference copy of the motor command, creates predictions in both modalities resulting in multisensory interactions in the context of action. The fact though that no interaction between action (passive/active) and type of stimuli (unimodal/bimodal) was observed in these studies suggested that self-generated action had no additive effect on MSI. This claim was also supported by Arikan et al.’s (2017) study that examined the window of subjective simultaneity of self-generated audiovisual stimuli with varying degrees of temporal asynchrony during voluntary and involuntary movements using the same paradigm (active versus passive button press). The action and its sensory consequences were either time contiguous or separated by one of two delays. When a delay between the action and the audiovisual stimuli was added the participants’ sensitivity for detecting audiovisual asynchrony was increased. However, when the researchers compared the two conditions, active button presses (voluntary movements) against passive button presses (involuntary movements), the results did not show differences in perceived simultaneity, supporting the claim of the previously mentioned studies for no additive effect of self-generated action on MSI capabilities. Desantis and Haggard (2016) examined the potential differences in the window of subjective simultaneity between an auditory and visual cue for predicted stimulus pairs (i.e., stimuli that followed a learning phase, so that the participants were familiarized with the exact pair) and for unpredicted pairs (i.e., untrained stimuli that the participants perceived for the first time). They also divided the experiment in trials where these stimuli were appearing after a self-generated action (button press) and trials where the stimuli were appearing after a visual cue (passive condition). The authors found differences in the window of subjective simultaneity between the conditions that the participants were prior trained for the stimuli and the conditions that the participants perceived the stimuli for the first time. However, there was no additive effect of the self- generated action (button press) versus the passive condition.

A clear effect of self-generated actions on MSI was reported in a recent study by Zierul and colleagues (2019). This study explored how self-generated actions affected the processing of multisensory stimuli in the spatial domain, focusing on the ventriloquist effect where visual stimuli can bias auditory localization. Specifically, this phenomenon describes the tendency to misjudge the location of a sound towards a corresponding visual cue. Participants were presented with audiovisual stimuli of varying temporal separation and onset predictability, that were either initiated by themselves (button press on a pointing stick) or externally generated (absence of button press). The authors found that self-initiation reduced the ventriloquist effect, suggesting that self-generated action results in more accurate MSI.

The study of Parsons et al. (2013) examined the effects of voluntary action on temporal binding and perceived synchrony between audiovisual stimuli by using an active (button press) versus passive (absence of button press) condition. Among other goals, the study used an SJ task to evaluate the perceived synchrony in the active and passive condition. The study revealed a decreased observed temporal asynchrony in the active as compared to the passive condition. This indicated that participants were more able to detect asynchronies between the stimuli when they were not correlated with the action, suggesting a lower MSI accuracy in the active condition. It should be noted, however, that this study was mainly designed to examine other cognitive processes, such as intentional binding, rather than the effects of self-generated action on MSI.

In conclusion these previous studies have produced conflicting results concerning the effects of self-generated action on MSI. The current study uses a novel paradigm to directly compare MSI between an active condition where multisensory stimuli are temporarily inked to the action that generated them, namely speech and a passive condition where the same multisensory stimuli are dissociated from the prior action that generated them. We utilized a version of the SJ task during speech (“talk versus listen task”). This task is linked with a specific mechanism of motor predictions and has high ecological validity of the sensory stimuli related with the motor action (Ford and Mathalon 2005; Kingyon et al. 2015). In the “talk” or active condition participants watched themselves on a computer monitor, while producing speech and listened to their speech echo, whereas in the “listen” or passive condition, participants watched a previously recorded video of themselves producing the same speech. In both conditions, SOAs between the auditory and visual stimuli were introduced and participants had to respond if they perceived the auditory (speech echo) and visual stimuli as synchronous or asynchronous. The responses determined the TBW of integration in the active and passive condition. We hypothesized that the TBW would be narrower in the active condition as compared to the passive condition, indicating more accurate MSI resulting from self – generated action.

## Methods

### Participants

G*Power (Faul et al. 2007) was used to perform a priori power analysis for a paired-samples t-test, comparing participants’ TBW estimation in the two conditions (i.e., active, passive). The effect size for this analysis was estimated based on Cohen’s (1988) guidelines. Based on a short pilot study, it was chosen that the best estimate of the true population standardized mean difference was δ = 0.95. This effect size estimate was entered into the power analysis with the following input parameters: a = 0.05, power = 0.95. The power analysis results suggested that a *N* = 14 was required to detect a difference of the mean TBW between the two experimental conditions with 95% probability. We, thus, recruited 9 male and 9 female participants between ages of 18 and 37 years old with a mean age of 28 years. The participants were selected using the following exclusion criteria: no prior history of central nervous system (CNS) disorder and not receiving any medication affecting the CNS function during testing. Participants were students that were recruited from the psychology department of Panteion University of Social and Political Sciences and the medical department of the National and Kapodistrian University of Athens. All participants agreed to participate in the study and signed a written informed consent. The study protocol was approved by the ethics committee of the University Mental Health Research Institute (protocol number: 231/05-04-2022) and the ethics committee of the Panteion University of Social and Political Sciences (protocol number: 14/10-5-2023). The experimental procedures conducted were in accordance with the ethical standards of the above committees and with the 1964 Helsinki declaration.

### Apparatus

**Fig. 2:**
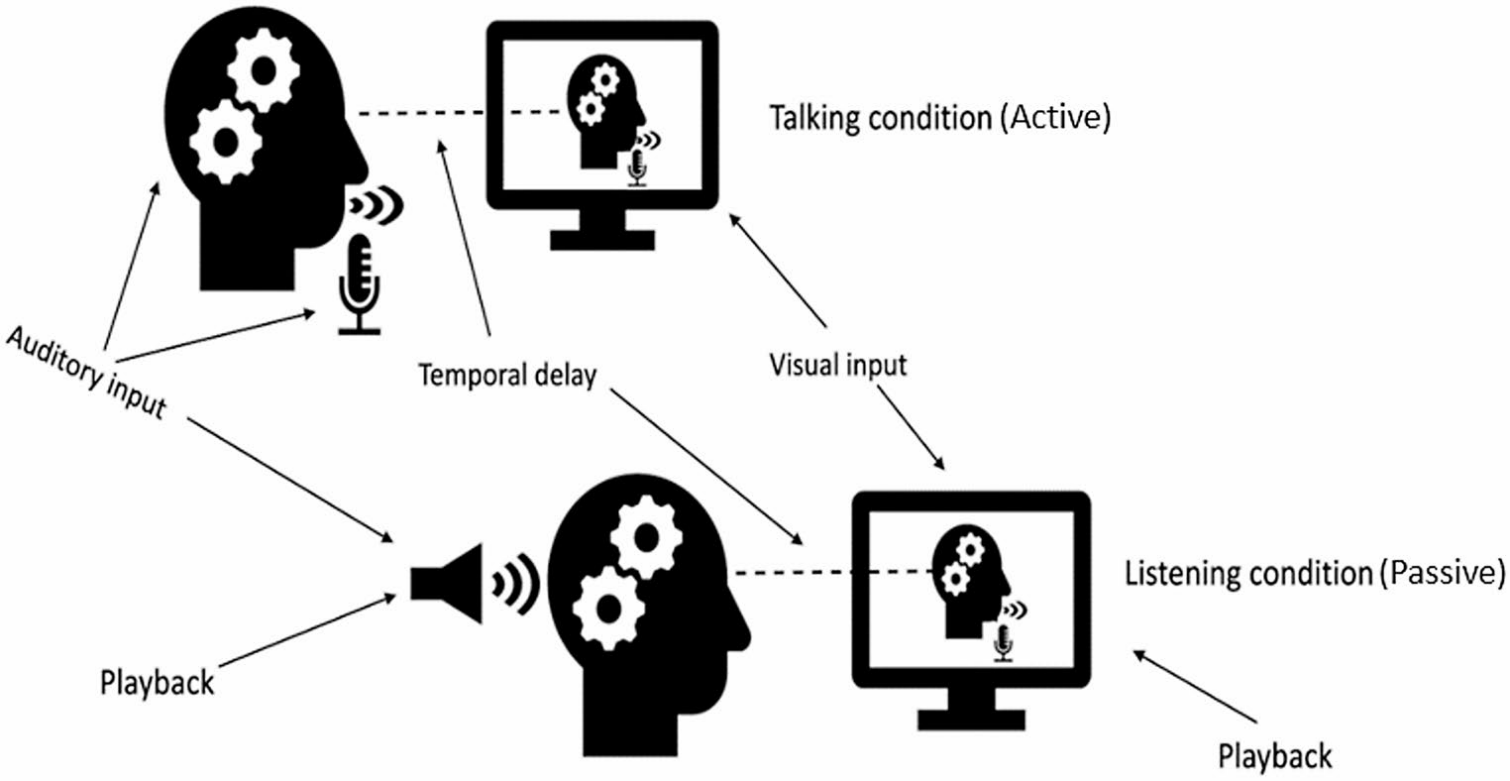
In the active condition, participants spoke via a microphone placed in front of them and they received the auditory input of the echo of their voice after some milliseconds, while they were seeing the image of themselves speaking also after some milliseconds after the onset of the lip movement. In the passive condition, participants received the prerecorded from the active condition auditory inputs (the syllable “ba” via the earphones) and the prerecorded from the active condition visual input (videos via the monitoring screen in front of them)

The experiment was programmed and run on vMix live video streaming software and E-prime (Psychology Software tools; Version 2.0) programming software. During the active condition, the auditory and visual stimuli were provided through vMix. vMix was running on a laptop (Lenovo Creator 5 i7–10750 H) that was projecting to an external monitor (24 – inch Dell S2417DG – LED Quad HD screen), 144 Hz refresh rate, set at 1024 × 768 resolution). As vMix does not allow the collection of responses, participants’ responses were collected via E-prime running on another computer (Intel (R) Core (TM) i5–4960) in the background). E-prime was also used for the passive condition, where both the visual and auditory stimuli were prerecorded from the active condition. A camera (Creative Live! Cam Sync 1080p V2) attached to the external monitor was capturing the face of the participants. Two microphones were used, one of them was the camera microphone and the other (Chronos response box integrated microphone) was placed in front of the participant and connected to the computer (see Fig. 2). The camera’s microphone was used to reproduce the echo of the voice of the participant and the other microphone to record the participant’s voice. The manual responses of the participants were collected via Chronos response box (Psychology Software tools) placed below their left hand. The Chronos response box was also connected to the voice recording microphone. Participants were receiving the auditory stimuli (echo of their voice) via wired earphones (Samsung Stereo Headset A500 3.5 mm) connected with the computer that vMix was running.

### Procedure

Participants were seated on a moveable chair in front of the monitor and the attached camera that was capturing their face, and they were instructed to remain as close to the recording microphone as possible during the whole duration of the experiment. Participants were also instructed prior to the onset of the experiment that they will perform an SJ task where they would have to judge if the echo of their voice would be synchronous with the image of themselves speaking. They were specifically instructed that the auditory stimulus would not be the real time voice that they were producing but the echo of the voice. Each trial in the active condition started with the presentation of a blank image in the computer monitor in front of the participant, which was followed by a cue (short beep sound) and the start of the monitoring of the participant who was instructed to speak the syllable “ba”.

We introduced variable auditory stimulus delays across trials, so that there would be a different delay in each trial between the time that the participant was speaking via the microphone and the time he/she was hearing the echo of his/her voice via the earphones. We also introduced variable visual delays across trials, so that there would be a different delay in each trial between the time that the participant was speaking and the time he/she was seeing him/herself speaking in the monitor. The audio and a visual delay from the onset of the speaking action ranged from 200 ms (minimum) to 600 ms (maximum). These delays reproduced a range of stimulus onset asynchronies (SOAs) between the auditory and the visual stimulus based on previous studies of MSI (Foss-Feig et al. 2010; Stevenson et al. 2014; Zvyagintsev et al. 2017). These SOAs were: ±67, 100, 167, 200, 267, 334, and 400 ms, where the negative sign indicates that the auditory stimulus was preceding the visual stimulus. For example, to create a -200ms SOA (with the auditory stimulus preceding the visual stimulus), the delay of the auditory stimulus from the onset of the speaking action in that trial would be 200 ms and for the visual stimulus 400ms. In half of the trials the auditory stimulus was preceding the visual stimulus (AV trials, negative SOA) and in the other half the visual stimulus was preceding the auditory stimulus (VA trials, positive SOA). The order of the trials was randomized in the beginning of the experiment.

In an initial calibration procedure, we measured the constant delays in the reproduction of the auditory stimuli from our system with Audacity software and of the visual stimuli with the help of a photodiode (Chronos response box integrated photo sensor) that was attached on the monitor and connected to the Chronos response box. The constant delay was 170 ms for the auditory stimuli with 5ms jitter and 150 ms for the visual stimuli with an 8 ms jitter. These constant delays were included in the calculations of delays to create the SOAs used in the experiment. In each trial, participants were instructed to respond by pressing a button if the echo of their voice speaking the syllable speech (auditory stimulus) and their self-image on the monitor speaking the syllable (visual stimulus) were synchronous and another button if they were asynchronous.

In the second, passive condition the same procedure was used as in the active condition except that the participants now were not performing an action to produce the sensory stimuli. The auditory stimulus was the prerecorded speech stimulus that was presented to the participants through the earphones and the visual stimulus was essentially the same prerecorded video of the participant speaking. Again, the participants had to press a button to indicate if the stimulus were synchronous or asynchronous. The active condition was performed before the passive condition to collect the recorded videos that would essentially be the sensory stimuli for the passive condition. However, we wanted to examine the possibility that the order of the conditions introduced a bias. Thus, in six participants, we changed the order of the conditions and conducted the passive condition first. This resulted in longer experimental time as it required an extra step where we recorded the speech videos and the sounds for the passive condition. We should note that participants did not perform the actual active condition of the task to prerecord the stimuli because that would add a potential training effect. We recorded the sounds and the videos separately, so that the participants were not familiarized at all with the task before they performed the passive condition.

The experiment lasted approximately two hours and included 4 blocks both for the active and the passive condition that lasted approximately 12 and 10 min, respectively, with 4 trials in each SOA per block, leading to a total of 16 trials per SOA, which resulted in a total of 224 trials. Two practice blocks corresponding to the active and passive conditions, respectively, were performed prior to the actual experimental blocks to familiarize the participants with the task.

For 7 participants, 1 (minimum) to 26 (maximum) trials of the active condition were rejected due to the microphone’s failure to detect the voice of the participant and they could not be used in the passive condition. We detected these trials by using the microphone that was connected to the Chronos box, which among other functions (e.g., collecting the actual responses) also detected a pseudo response with a single timestamp each time the participant spoke. Four of our participants also performed three instead of four blocks in the active condition and one participant performed three blocks in the active and two in the passive condition. The data of this participant were excluded from the analysis because they were considered insufficient (almost half of the experiment conducted).

The fact that the participants were hearing their voice twice in the active condition (real speech and echo) could generate confusing sensory effects. To test the impact of this confusing effect of sensory stimulation in the active condition we retested 8 out of 18 participants 6 months after the initial experimentation on a variation of the passive condition in which the participants heard their voice two times. The time interval in each trial between the first and the second time the participants were hearing their voice were adjusted to be identical as the distances between the time the participants were speaking and hearing the echo of their voice in the active condition.

### Analysis

The analysis was conducted for 17 participants. Each participants’ responses in each condition were used to compute the TBW for MSI based on the method used by Stevenson et al. (2012). More specifically, we computed the rate of perceived simultaneity for each condition and SOA by calculating the percentage of trials in which the individual reported simultaneous presentation. Subsequently, two psychometric sigmoid functions were fitted to the rates of perceived simultaneity for the auditory-first and visual-first presentations, respectively. The sigmoid functions were fitted to the auditory first (AV) and visual first (VA) windows and included all the SOAs. The best-fit sigmoids were generated using the glmfit function in MATLAB. Following this, the individual AV and VA side of the TBWs were estimated for each participant as the SOA at which the best-fit sigmoid’s y-value was equal to a 50% rate of perceived simultaneity. Lastly, the group TBWs were calculated and plotted by taking the arithmetic mean of the respective AV and VA side of the TBW from all 17 participants. The script used to calculate the TBW was based on Notter and Murray’s (2017) method, but with the following change: our script did not split the data into two equal big parts, but it spliced it according to the onset asynchronies to avoid small potential biases from the few rejected trials in the active condition.

First, we conducted a repeated measures ANOVA with within factors the condition (active vs. passive) and the order of sensory stimuli presentation (AV vs. VA side of the window).

We then used multiple paired samples t-tests to compare the AV, VA part and total TBW between the active and passive conditions. This also allowed us to compare the AV, VA and whole TBW separately and include effect sizes (calculated using the Cohen’s d). In addition, we compared the maximum points of the TBW curves between the active and passive conditions using paired sample t-test. The maximum point of the TBW curve is a measure of the participants confidence of the SJ. We also used paired sample t-tests to compare the VA, AV and whole TBW values of the variant passive condition and the active condition for the eight participants that performed the variant passive condition. Finally, we conducted another set of paired sample t-tests comparing the average values of the VA, AV and whole TBW between active and passive condition for the six participants where the order of presentation was reversed (passive then active).

## Results

**Fig. 3:**
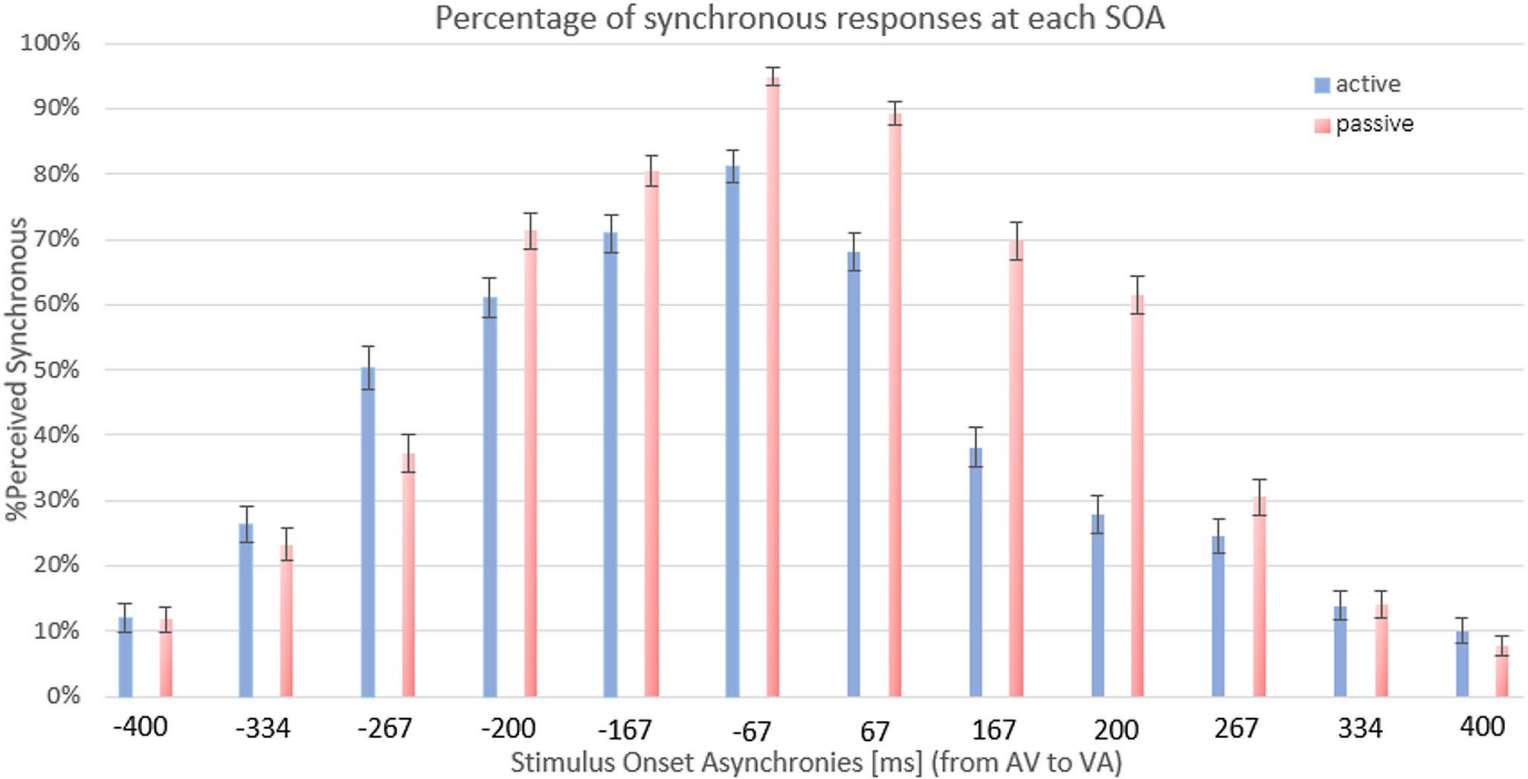
Mean percentage of responses (bars indicate SE of the mean) where participants judged that the echo of their voice and the image of themselves speaking were synchronous (% perceived synchronous, y axis) at each stimulus onset asynchrony (SOA, x axis). The blue bars depict the percentage of synchrony for the active condition and the red bars the percentage of synchrony for the passive condition. These values were used to estimate the curves of temporal binding window as depicted in Fig. 4

Participants responded that the echo of their voice speaking the syllable speech (auditory stimulus) and their self-image on the monitor speaking the syllable (visual stimulus) were synchronous (% of perceived synchrony) more often in the passive condition than the active as seen in Fig. 3. This effect was prominent when the visual stimulus preceded the auditory one (VA order, positive SOAs).

**Fig. 4:**
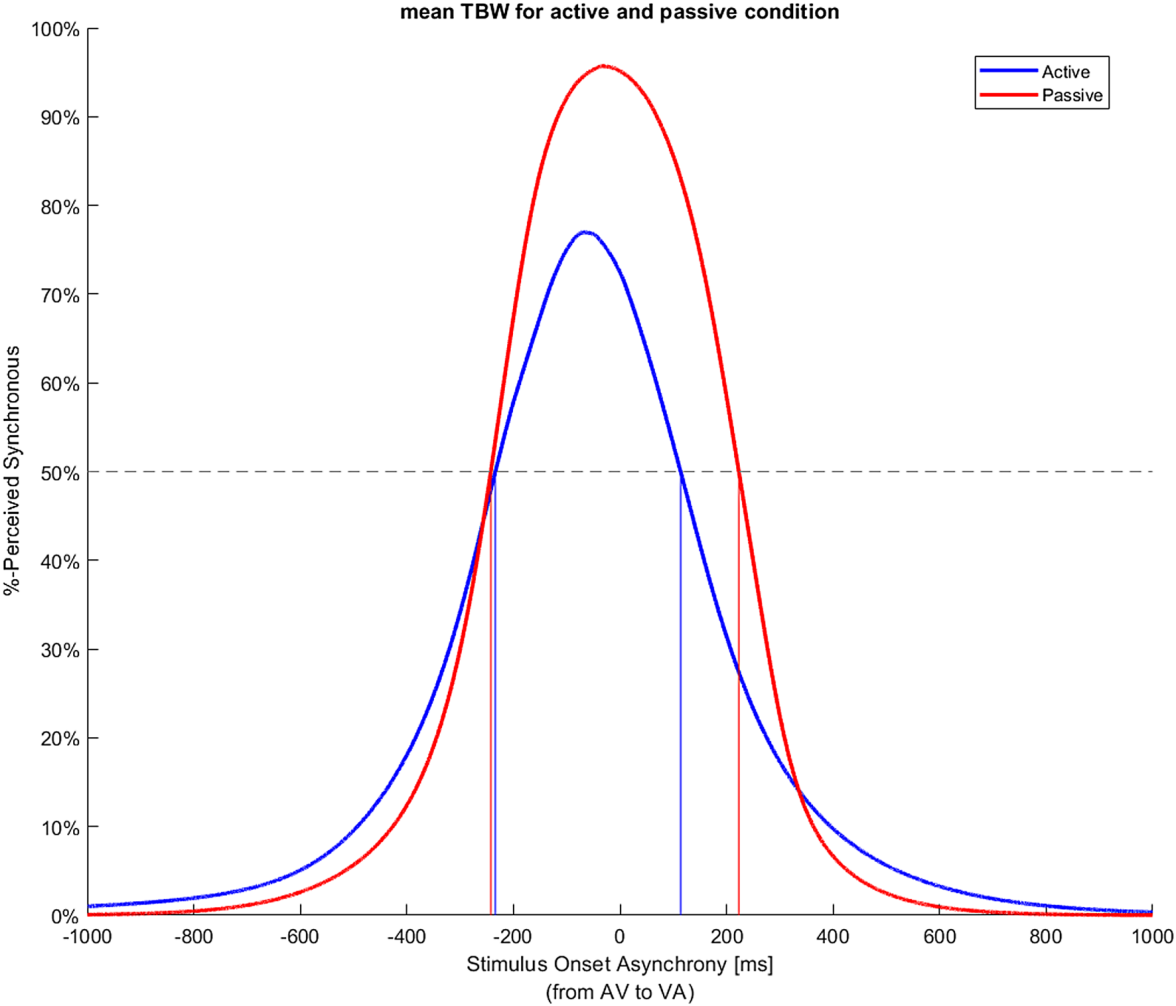
The average temporal binding window in the active (blue) and passive (red) condition. The x-axis represents the stimulus onset asynchronies (SOAs) where the negative ones indicate those where the auditory stimulus leads (AV), and the positive ones indicate those where the visual stimulus leads (VA). The y-axis represents the percentage of the synchrony reported by the participants in each SOA. The SOAs in the x-axis where the percentage of synchrony is above 50% represent the limits of the temporal binding window (TBW) for the AV and VA conditions, respectively. These limits are depicted with blue vertical lines for the active condition and red vertical lines for the passive condition. The area within those limits depicts the TBW for each condition. The curves were plotted using the arithmetic mean for the AV and VA part of the TBW from all 17 participants for the active and passive condition respectively

On average, the AV part of TBW was 240.76 ms (SD: 100.71 ms) for the active condition and 259 ms (SD: 79.13 ms) for the passive condition and the VA part of the TBW was 119.53 ms (SD: 91.45 ms) for the active condition and 222.41 ms (SD: 82.97 ms) for the passive condition. The total TBW was 360.24 ms (SD: 133.78 ms) for the active condition and 481.53 ms (SD: 122.46 ms) for the passive condition (Fig. 4). The repeated measures ANOVA revealed a statistically significant main effect of Condition (Active vs. Passive), (F _(1, 16)_ = 30.005, *p* < 0.001, eta_p^2^ = 0.652). The main effect of order (AV vs. VA) was also significant (F _(1, 16)_ = 9.73, *p* = 0.007, eta_ p^2^ = 0.378). Finally, a significant interaction condition by order effect was also observed (F _(1, 16)_ = 7.050, *p* = 0.017, eta_ p^2^ = 0.306).

**Fig. 5:**
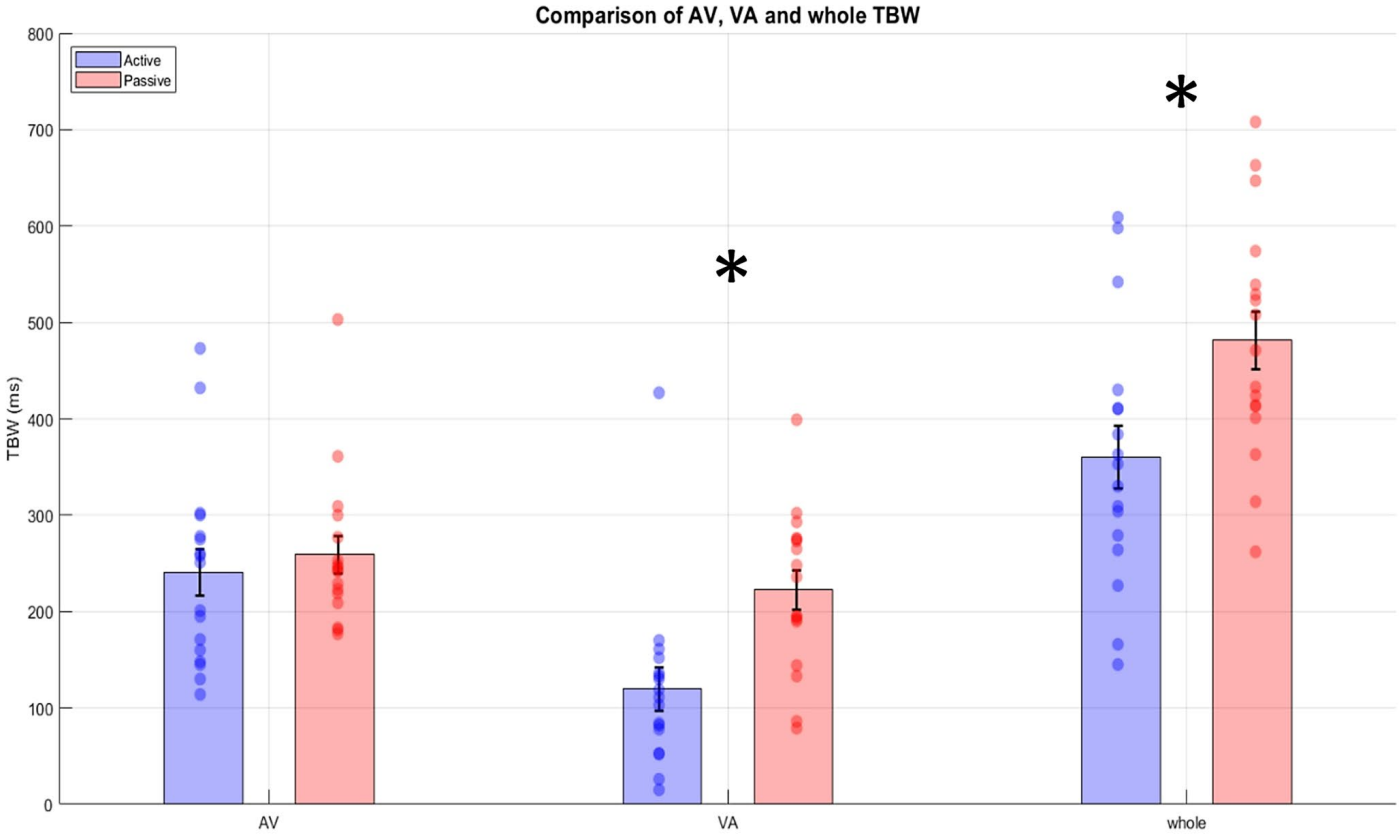
Pairwise comparisons between active and passive condition for the AV, VA part of the TBW and whole TBW. The bars represent the mean for each condition and the lines on top them the SEMs. The dots within each bar represent the individual values of each participant. Statistically significant differences are marked with an asterisk

Paired samples t tests revealed that the average AV part of the TBW in the active condition was not significantly different from the AV part of the TBW in the passive condition, (t_(16)_ = 0.847, *p* = 0.409, Cohen’s d = 0.206) (Fig. 5), while the average VA part of the TBW in the active condition was significantly narrower than in the passive, (t_(16)_ = 6.046, *p* < 0.001, Cohen’s d = 1.466) (Fig. 5). The total TBW was also significantly narrower in the active condition compared to the passive condition (t_(16)_ = 5.491, *p* < 0.001, Cohen’s d = 1.332) (Fig. 5).

In the subset of participants that performed the task in the reverse order the average AV part of the TBW was 296.67 ms (SD: 130.04 ms) for the active condition and 297.33 ms (SD:104.76 ms) for the passive condition, and the VA part of the TBW was 105.17 ms (SD: 21.76 ms) for the active condition and 192.17 ms (SD: 71.33 ms) for the passive condition. The total TBW was 401.67 ms (SD: 141.8 ms) for the active condition and 489.83 ms (SD: 106.94 ms) for the passive condition. The paired samples t-tests between active and passive condition for the AV, VA and TBW for these 6 participants showed the same significant effects that were found for the whole dataset. Specifically, the AV part of the TBW in the active condition was not significantly different from the AV part of the TBW in the passive condition, (t_(5)_ = 0.0216, *p* = 0.984, Cohen’s d = 0.009), while the average VA part of the TBW in the active condition was significantly narrower than in the passive, (t_(5)_ = 3.04, *p* = 0.029, Cohen’s d = 1.24). The total TBW was also significantly narrower in the active condition compared to the passive condition (t_(5)_ = 3.13, *p* = 0.026, Cohen’s d = 1.28).

We observed a shift in the TBW towards the auditory lead in the active condition compared to the passive condition (Fig. 4). A paired samples t-test between the AV and the VA part of the TBW showed a significantly larger TBW when the auditory stimulus was preceding the visual stimulus (AV) as compared to when the visual stimulus was presented before the auditory stimulus (VA), t_(16)_ = 3.618, *p* = 0.002 Cohen’s d = 0.877, in the active condition, while no statistically significant differences were found in the passive condition, t_(16)_ = 1.419, *p* = 0.175, Cohen’s d = 0.344. Finally, the maximum point of the TBW curve was significantly smaller in the active than in the passive condition, t_(16)_ = 5.879, *p* < 0.001, Cohen’s d = 1.426, suggesting that the participants were more confident about their responses in the passive condition compared to the active condition.

The individual values of the AV, VA and whole TBW for the passive condition with one auditory stimulus and two auditory stimuli are presented in supplementary Table 1. The average AV part of the TBW was 273.12ms (SD: 109.68 ms) for the passive condition with one auditory stimulus and 279.87 ms (SD:123.75 ms) for the passive condition with two auditory stimuli. The average VA part of the TBW was 165.12 ms (SD: 71.84 ms) for the passive condition with one auditory stimulus and 167.75 ms (SD:78.88 ms) for the passive condition with two auditory stimuli. Lastly, the whole TBW was calculated at 438.38 ms (SD: 145.34 ms) for the passive condition with one auditory stimulus and at 447.5 ms (SD: 159.2 ms) for the passive condition with two auditory stimuli. The paired samples t-test between the variant passive condition with two auditory stimuli and the original active condition showed the same significant effects as were observed for the original dataset. Specifically, there was a significant difference between the active and passive condition for the VA part of the temporal binding window (t_(7)_ = 3.37 *p* = 0.012, Cohen’s d = 1.193) and a significant difference for the whole TBW (t_(7)_ = 3.35 *p* = 0.012, Cohen’s d = 1.183). For the AV part of the TBW the difference was found at the limit of statistical significance (t_(7)_ = 2.36 *p* = 0.05, Cohen’s d = 0.836).

## Discussion

The present study investigated the potential effect of self-generated action in MSI by examining the difference between the TBW when both multisensory stimuli are self-generated versus when they are passively received by the participants. We showed that the TBW of MSI was smaller in the active condition compared to the passive condition, suggesting more efficient MSI in the case where multisensory stimuli arise from self-generated action, namely speech. Furthermore, the findings revealed that the TBW was auditory shifted in the active condition, while it was symmetric in the passive condition, and this explains why the differences between the two TBWs are most prominent for the VA side of the window. Finally, a larger rate of confidence was observed for responses in the passive condition compared to the active condition.

The first and most prominent finding of this study that confirms our hypothesis is the narrower TBW observed when multisensory stimuli are generated by the participants’ own actions in the paradigm of speech indicating increased accuracy of MSI. The results of this study are in accordance with the results of the study of Zierul et al. 2019. In that study the ventriloquist effect was reduced when stimulus presentation was self-initiated (active condition) compared to externally initiated (passive condition). The reduction of the illusory effect corresponds to more accurate MSI in space in the same fashion as reduced TBW corresponds to more accurate MSI in time.

The studies of van Kemenade et al. (2016) and Straube et al. (2017) were not designed to address directly the effect of self-generated action on MSI. In both studies, the detection of a temporal delay between action and stimulus presentation was enhanced for active as compared to passive conditions but this phenomenon was observed in both bimodal and unimodal stimuli with no significant interaction between the condition type (active, passive) and the type of stimulus presented (unimodal, bimodal). Two studies examined directly the effect of self-generated action on MSI and found no change in MSI capacity comparing the active condition (induced by self-generated action) to the passive condition (Arikan et al. 2017; Desantis and Haggard 2016). Finally, the study of Parson et al. (2013) showed the opposite effect of self-generated action on MSI. The study hypothesized that two stimuli originating from a common source (a motor action in this case) are more likely to be interpreted as simultaneous with another. Our study results argue against this hypothesis by showing a higher probability of detecting asynchronies when the sensory stimuli follow a self-initiated movement as compared to being initiated without the participant’s control.

In addition, the analysis that we performed on the maximum point of the TBW curve reveals that the participants were more confident of their responses in the passive condition. This could be described as a paradoxical finding of our study as we expected participants to be more confident in the active condition at all SOAs given the better MSI capacity reflected in the narrower TBW. Our results might relate to the higher complexity of the task in the active condition. More specifically, participants had to detect synchrony between the auditory and the visual stimulus in both experimental conditions, but in the active condition this detection was preceded by the generation of the stimuli themselves which could add an extra degree of task complexity.

In our study, we observed an increase in MSI accuracy (measured as decrease in the width of the TBW of integration) for the active versus passive condition, which was mainly focused on the VA side of the window of integration. The major difference between our study and previous studies was the use of a novel paradigm which has a high degree of ecological validity as both stimuli that were being presented were related with our actions very often in our everyday lives (e.g., we hear our voice, or even the echo of our voice, very often and we see ourselves speaking via a camera or a mirror in a frequent basis as well). We speculate that the paradigm that we used could be more fit to study a potential link between sensory predictions and MSI because speech is a process closely linked with both cognitive processes. This paradigm could also be used to study the effect of the acting agent on MSI. It has been shown that sensory attenuation of self-generated unimodal sensory signals is lower compared to similar sensory stimuli generated by another agent (Sato 2008, 2009; Pyasik et al. 2021). To explore the difference between self and other we could use a modified version of our paradigm to test MSI in two conditions, the passive condition of the current design using previously recorded self- generated speech and another passive condition using the recorded audiovisual presentation of the same speech produced by another person.

The smaller TBW that we observed in the active condition could be explained by faciliatory effect of top-down prediction of the timing of multisensory stimuli via the transmission of efference copies. This interpretation is supported by previous research showing that top-down factors can modulate the sensory processing of unimodal but also bimodal stimuli (van Kemenade et al. 2016). It is also consistent with the studies of Ford and Mathalon (2005; 2019), that used the paradigm of speech to show that the transmission of efference copies at the onset of the lip movement (onset of speech) allows the prediction of the sensory consequences of the speech action. The same process might explain the results of the current study. More specifically the onset of the lip movement (onset of speech) might facilitate the generation of sensory predictions for both the auditory (echo of the voice) and the visual (view of the face speaking) stimuli via the transmission of efference copies. Unlike previous studies that used button press as the action to trigger unrelated auditory and visual stimulus presentation, our paradigm uses an action (speech) that triggers auditory (echo of our voice) and visual (see ourselves talking in a mirror) stimuli with which we are overly familiarized thus reinforcing the prediction of these outcomes via the transmission of efference copies. Our brains are trained to predict and suppress the auditory outcome of speech (hearing ourselves speaking) since infancy (Behroozmad and Larson, 2011). Similarly, the visual outcome of speech is well learned (e.g. talking to a mirror) and this efferent copy information has been shown to play a vital role in visual self – recognition (Tsakiris et al. 2005). In summary we hypothesize that the natural action of speech results in the robust transmission of audiovisual efference copies of the speech act which is not the case for a button press linked to abstract audiovisual stimuli that people are not trained to link with this action. These efference copies then facilitate multisensory integration of speech related audiovisual sensory stimuli.

Another possible explanation for the smaller TBW in the active condition is that it might reflect increased attentional resources being allocated to the task, leading to more efficient MSI. Especially in the visual domain, participants might be more focused on specific facial characteristics (i.e., lip movements), when they are actively generating the sensory stimuli. This may result in faster and more accurate processing of the stimuli. The current interpretation is in line with previous studies that have suggested that top-down visuospatial attention during speech can alter MSI (Senkowski et al. 2008; Talsma and Woldorff 2005). Former research exploring potential links between attention and prediction have revealed that these cognitive processes can interact with each other. For instance, attention has been suggested to enhance the discrimination between expected and unexpected sounds (Jiang et al. 2013). This observation might also indicate a differentiation between general gating during actions and precise predictions leading to sensory suppression (Schröger et al. 2015).

In the current study we also observed an auditory shift of the TBW in the active condition that was not present in the passive condition. This shift resulted in a very large difference of the two conditions on the VA side of the TBW while there was no difference on the AV side. A hypothesis for the explanation of the auditory shift of the TBW could be that it was caused by the distractive effect of the double auditory stimulation in the trials when the auditory stimulus was leading the visual stimulus. In these trials, the echo of the voice was heard in very close proximity to the actual hearing of one’s voice that might lead to a distraction effect. We explicitly tested this hypothesis in the additional passive condition. We showed that when participants were hearing their voice two times in proximity, mimicking the auditory stimulation of the active condition, the TBW was identical to that observed in the original passive condition and the same effect was found between the active and passive condition. Thus, the auditory shifted TBW in the active condition cannot be attributed to the distracting effect of the double auditory stimulus. We cannot rule out a more complex interaction of MSI with the preceding motor action leading to this effect. Future research is needed to identify the exact cause for this asymmetry in the TBW in the active condition in the current paradigm.

## Conclusion

The present study contributes to our understanding of the mechanisms underlying MSI. We highlight the role of self-generated input in the process of MSI, specifically when stimuli used in the active condition have a high ecological validity (listening and seeing oneself speaking). We hypothesize that the origin of enhanced MSI accuracy we observed in the active condition might relate to self-generated predictions via efference copy transmissions to create expectations about the timing of the multisensory stimuli or alternatively to allocation of increased attentional resources or to a combination of these effects. Future experiments could test these hypotheses probing the neurophysiological correlates of MSI in this paradigm with concurrent EEG measurements.

## Electronic supplementary material

Below is the link to the electronic supplementary material.

Supplementary Material 1

## Data Availability

The data collected for this study are available from the authors upon request.

## References

[CR1] Arikan BE, van Kemenade BM, Straube B, Harris LR, Kircher T (2017) Voluntary and involuntary movements widen the window of subjective simultaneity. I-Perception 8(4):204166951771929. 10.1177/2041669517719297 10.1177/2041669517719297 PMC5528186 28835813

[CR2] Behroozmand R, Larson CR (2011) Error-dependent modulation of speech-induced auditory suppression for pitch-shifted voice feedback. BMC Neurosci 12(1). 10.1186/1471-2202-12-54 10.1186/1471-2202-12-54 PMC3120724 21645406

[CR4] Blakemore S, Rees G, Frith CD (1998a) How do we predict the consequences of our actions? A functional imaging study. Neuropsychologia 36(6):521–529. 10.1016/s0028-3932(97)00145-0 9705062 10.1016/s0028-3932(97)00145-0

[CR5] Blakemore S-J, Wolpert DM, Frith CD (1998b) Central cancellation of self-produced tickle sensation. Nat Neurosci 1(7):635–640. 10.1038/2870 10196573 10.1038/2870

[CR3] Blakemore S-J, Frith CD, Wolpert DM (1999) Spatio-temporal prediction modulates the perception of self-produced stimuli. J Cogn Neurosci 11(5):551–559. 10.1162/089892999563607 10511643 10.1162/089892999563607

[CR6] Blakemore S-J, Wolpert D, Frith C (2000) Why Canʼt You Tickle Yourself? NeuroReport 11(11). 10.1097/00001756-200008030-00002 10.1097/00001756-200008030-00002 10943682

[CR7] Canzoneri E, Magosso E, Serino A (2012b) Dynamic sounds capture the boundaries of peripersonal space representation in humans. PLoS ONE 7(9). 10.1371/journal.pone.0044306 10.1371/journal.pone.0044306 PMC3460958 23028516

[CR57] Cohen J (1988) The effect size. In Statistical power analysis for the behavioral sciences. Routledge, Abingdon, pp 77–83

[CR8] Conrey B, Pisoni DB (2006a) Auditory-visual speech perception and synchrony detection for speech and nonspeech signals. J Acoust Soc Am 119(6):4065–4073. 10.1121/1.2195091 16838548 10.1121/1.2195091 PMC3314884

[CR9] De Niear MA, Gupta PB, Baum SH, Wallace MT (2018) Perceptual training enhances Temporal acuity for multisensory speech. Neurobiol Learn Mem 147:9–17. 10.1016/j.nlm.2017.10.016 29107704 10.1016/j.nlm.2017.10.016

[CR10] Desantis A, Haggard P (2016) Action-outcome learning and prediction shape the window of simultaneity of audiovisual outcomes. Cognition 153:33–42. 10.1016/j.cognition.2016.03.009 27131076 10.1016/j.cognition.2016.03.009

[CR11] Diederich A, Colonius H (2009) Crossmodal interaction in speeded responses: time window of integration model. Prog Brain Res 119–135. 10.1016/s0079-6123(09)01311-9 10.1016/S0079-6123(09)01311-9 19477335

[CR12] Faul F, Erdfelder E, Lang A-G, Buchner A (2007) G*Power 3: A flexible statistical power analysis program for the social, behavioral, and biomedical sciences. Behav Res Methods 39(2):175–191. 10.3758/bf03193146 17695343 10.3758/bf03193146

[CR13] Ford JM, Mathalon DH (2005) Corollary discharge dysfunction in schizophrenia: can it explain auditory hallucinations? Int J Psychophysiol 58(2–3):179–189. 10.1016/j.ijpsycho.2005.01.014 16137779 10.1016/j.ijpsycho.2005.01.014

[CR14] Ford JM, Mathalon DH (2019) Efference copy, corollary discharge, predictive coding, and psychosis. Biol Psychiatry: Cogn Neurosci Neuroimaging 4(9):764–767. 10.1016/j.bpsc.2019.07.005 31495399 10.1016/j.bpsc.2019.07.005

[CR15] Foss-Feig JH, Kwakye LD, Cascio CJ, Burnette CP, Kadivar H, Stone WL, Wallace MT (2010) An extended multisensory Temporal binding window in autism spectrum disorders. Exp Brain Res 203(2):381–389. 10.1007/s00221-010-2240-4 20390256 10.1007/s00221-010-2240-4 PMC2871100

[CR16] Galigani M, Castellani N, Donno B, Franza M, Zuber C, Allet L, Garbarini F, Bassolino M (2020b) Effect of tool-use observation on metric body representation and peripersonal space. Neuropsychologia 148:107622. 10.1016/j.neuropsychologia.2020.107622 32905815 10.1016/j.neuropsychologia.2020.107622

[CR17] Horsfall RP, Wuerger SM, Meyer GF (2021) Narrowing of the audiovisual Temporal binding window due to perceptual training is specific to high visual intensity stimuli. I-Perception 12(1):204166952097867. 10.1177/2041669520978670 10.1177/2041669520978670 PMC7897829 33680418

[CR18] Jack BN, Pelley L, Han ME, Harris N, Spencer AWF, K. M., Whitford TJ (2019) Inner speech is accompanied by a temporally-precise and content-specific corollary discharge. NeuroImage 198:170–180. 10.1016/j.neuroimage.2019.04.038 31002966 10.1016/j.neuroimage.2019.04.038

[CR19] Jiang J, Summerfield C, Egner T (2013) Attention sharpens the distinction between expected and unexpected percepts in the visual brain. J Neurosci 33(47):18438-18447. 10.1523/jneurosci.3308-13.2013. 10.1523/JNEUROSCI.3308-13.2013 PMC3834051 24259568

[CR20] Kingyon J, Behroozmand R, Kelley R, Oya H, Kawasaki H, Narayanan NS, Greenlee JDW (2015) High-gamma band fronto-temporal coherence as a measure of functional connectivity in speech motor control. Neuroscience 305:15–25. 10.1016/j.neuroscience.2015.07.069 26232713 10.1016/j.neuroscience.2015.07.069 PMC4747053

[CR21] Kostaki M, Vatakis A (2018) Temporal order and synchrony judgments: A primer for students. Timing time Perception: Procedures Measures Appl 233–262. 10.1163/9789004280205_012

[CR22] Lee H, Noppeney U (2014) Music expertise shapes audiovisual Temporal integration windows for speech, sinewave speech, and music. Front Psychol 5. 10.3389/fpsyg.2014.00868 10.3389/fpsyg.2014.00868 PMC4124486 25147539

[CR23] McGovern DP, Roudaia E, Newell FN, Roach NW (2016) Perceptual learning shapes multisensory causal inference via two distinct mechanisms. Sci Rep 6(1). 10.1038/srep24673 10.1038/srep24673 PMC4835789 27091411

[CR24] Mcgurk H, Macdonald J (1976) Hearing lips and seeing voices. Nature 264(5588):746–748. 10.1038/264746a0 1012311 10.1038/264746a0

[CR25] Noel J-P, Paredes R, Terrebonne E, Feldman JI, Woynaroski T, Cascio CJ, Seriès P, Wallace MT (2022) Inflexible updating of the self-other divide during a social context in autism: psychophysical, electrophysiological, and neural network modeling evidence. Biol Psychiatry: Cogn Neurosci Neuroimaging 7(8):756–764. 10.1016/j.bpsc.2021.03.013 33845169 10.1016/j.bpsc.2021.03.013 PMC8521572

[CR26] Notter MP, Murray MM (2017) Temporal binding window scripts: A lightweight matlab tool to analyse the Temporal binding window in a multisensory integration study Zenodo.10.5281/zenodo.815876.

[CR27] Parlikar R, Bose A, Venkatasubramanian G (2019) Schizophrenia and corollary discharge: A neuroscientific overview and translational implications. Clin Psychopharmacol Neurosci 17(2):170-182. 10.9758/cpn.2019.17.2.170. 10.9758/cpn.2019.17.2.170 PMC6478093 30905117

[CR28] Parsons BD, Novich SD, Eagleman DM (2013) Motor-sensory recalibration modulates perceived simultaneity of cross-modal events at different distances. Front Psychol 4. 10.3389/fpsyg.2013.00046 10.3389/fpsyg.2013.00046 PMC3582016 23549660

[CR29] Powers AR, Hillock AR, Wallace MT (2009) Perceptual training narrows the Temporal window of multisensory binding. J Neurosci 29(39):12265 -12274. 10.1523/jneurosci.3501-09.2009. 10.1523/JNEUROSCI.3501-09.2009 PMC2771316 19793985

[CR30] Pyasik M, Ronga I, Burin D, Salatino A, Sarasso P, Garbarini F, Ricci R, Pia L (2021) I’m a believer: illusory self-generated touch elicits sensory Attenuation and somatosensory evoked potentials similar to the real self-touch. NeuroImage 117727. 10.1016/j.neuroimage.2021.117727 10.1016/j.neuroimage.2021.117727 33434613

[CR31] Sato A (2008) Action observation modulates auditory perception of the consequence of others’ actions. Conscious Cogn 17(4):1219 -1227. 10.1016/j.concog.2008.01.003. 10.1016/j.concog.2008.01.003 18299207

[CR32] Sato A (2009) Both motor prediction and conceptual congruency between preview and action-effect contribute to explicit judgment of agency. Cognition 110(1):74 -83. 10.1016/j.cognition.2008.10.011. 10.1016/j.cognition.2008.10.011 19038380

[CR33] Schröger E, Marzecová A, SanMiguel I (2015) Attention and prediction in human audition: A lesson from cognitive psychophysiology. Eur J Neurosci 5641–664. 10.1111/ejn.12816. 41 10.1111/ejn.12816 PMC4402002 25728182

[CR34] Scott M (2013) Corollary discharge provides the sensory content of inner speech. Psychol Sci 91824–1830. 10.1177/0956797613478614. 24 10.1177/0956797613478614 23846719

[CR35] Senkowski D, Saint-Amour D, Gruber T, Foxe JJ (2008) Look who’s talking: the deployment of visuo-spatial attention during multisensory speech processing under noisy environmental conditions. NeuroImage 43(2):379 -387. 10.1016/j.neuroimage.2008.06.046. 10.1016/j.neuroimage.2008.06.046 PMC2596295 18678262

[CR36] Shams L, Kamitani Y, Shimojo S (2000) What you see is what you hear. Nature 408(6814):788–788. 10.1038/35048669 11130706 10.1038/35048669

[CR37] Stein B, Meredith M (1993) The merging of the senses. J Cogn Neurosci 5(3):373–374. 10.1162/jocn.1993.5.3.373 23972225 10.1162/jocn.1993.5.3.373

[CR38] Stein BE, Stanford TR, Ramachandran R, Perrault TJ, Rowland BA (2009) Challenges in quantifying multisensory integration: alternative criteria, models, and inverse effectiveness. Exp Brain Res 198:2–3. 10.1007/s00221-009-1880-8 10.1007/s00221-009-1880-8 PMC3056521 19551377

[CR39] Stein BE, Stanford TR, Rowland BA (2014) Development of multisensory integration from the perspective of the individual neuron. Nat Rev Neurosci 15(8):520–535. 10.1038/nrn3742 25158358 10.1038/nrn3742 PMC4215474

[CR41] Stevenson RA, Wallace MT (2013) Multisensory Temporal integration: task and stimulus dependencies. Exp Brain Res 227(2):249–261. 10.1007/s00221-013-3507-3 23604624 10.1007/s00221-013-3507-3 PMC3711231

[CR42] Stevenson RA, Zemtsov RK, Wallace MT (2012) Individual differences in the multisensory Temporal binding window predict susceptibility to audiovisual illusions. J Exp Psychol Hum Percept Perform 38(6):1517–1529. 10.1037/a0027339 22390292 10.1037/a0027339 PMC3795069

[CR40] Stevenson RA, Ghose D, Fister JK, Sarko DK, Altieri NA, Nidiffer AR, Kurela LR, Siemann JK, James TW, Wallace MT (2014) Identifying and quantifying multisensory integration: A tutorial review. Brain Topogr 27(6):707–730. 10.1007/s10548-014-0365-7 24722880 10.1007/s10548-014-0365-7

[CR43] Straube B, van Kemenade BM, Arikan BE, Fiehler K, Leube DT, Harris LR, Kircher T (2017) Predicting the multisensory consequences of one’s own action: bold suppression in auditory and visual cortices. PLoS ONE 12(1). 10.1371/journal.pone.0169131 10.1371/journal.pone.0169131 PMC5218407 28060861

[CR44] Talsma D, Woldorff MG (2005) Selective attention and multisensory integration: multiple phases of effects on the evoked brain activity. J Cogn Neurosci 17(7):1098–1114. 10.1162/0898929054475172 16102239 10.1162/0898929054475172

[CR45] Tsakiris M, Haggard P, Franck N, Mainy N, Sirigu A (2005) A specific role for efferent information in self-recognition. Cognition 96(3):215–231. 10.1016/j.cognition.2004.08.002 15996559 10.1016/j.cognition.2004.08.002

[CR46] van Kemenade BM, Arikan BE, Kircher T, Straube B (2016) Predicting the sensory consequences of one’s own action: first evidence for multisensory facilitation. Atten Percept Psychophys 78(8):2515–2526. 10.3758/s13414-016-1189-1 27515031 10.3758/s13414-016-1189-1

[CR48] Vatakis A, Spence C (2006) Audiovisual synchrony perception for music, speech, and object actions. Brain Res 1111(1):134–142. 10.1016/j.brainres.2006.05.078 16876772 10.1016/j.brainres.2006.05.078

[CR50] Vatakis A, Spence C (2008) Evaluating the influence of the ‘unity assumption’ on the Temporal perception of realistic audiovisual stimuli. Acta Psychol 127(1):12–23. 10.1016/j.actpsy.2006.12.002 10.1016/j.actpsy.2006.12.002 17258164

[CR49] Vatakis A, Spence C (2010) Audiovisual Temporal integration for complex speech, object-action, animal call, and musical stimuli. Multisensory Object Percept Primate Brain 95–121. 10.1007/978-1-4419-5615-6_7

[CR47] Vatakis A, Maragos P, Rodomagoulakis I, Spence C (2012) Assessing the effect of physical differences in the articulation of consonants and vowels on audiovisual Temporal perception. Front Integr Nuerosci 6. 10.3389/fnint.2012.00071 10.3389/fnint.2012.00071 PMC3461522 23060756

[CR51] Wallace MT, Stevenson RA (2014) The construct of the multisensory Temporal binding window and its dysregulation in developmental disabilities. Neuropsychologia 64:105–123. 10.1016/j.neuropsychologia.2014.08.005 25128432 10.1016/j.neuropsychologia.2014.08.005 PMC4326640

[CR52] Wolpert DM, Flanagan JR (2001) Motor prediction. Curr Biol 11(18). 10.1016/s0960-9822(01)00432-8 10.1016/s0960-9822(01)00432-8 11566114

[CR53] Wurtz RH, Sommer MA (2004) Identifying corollary discharges for movement in the primate brain. Prog Brain Res 47–60. 10.1016/s0079-6123(03)14403-2 10.1016/S0079-6123(03)14403-2 14650839

[CR54] Zhu H, Tang X, Chen T, Yang J, Wang A, Zhang M (2023) Audiovisual illusion training improves multisensory Temporal integration. Conscious Cogn 109:103478. 10.1016/j.concog.2023.103478 36753896 10.1016/j.concog.2023.103478

[CR55] Zierul B, Tong J, Bruns P, Röder B (2019) Reduced multisensory integration of self-initiated stimuli. Cognition 182:349–359. 10.1016/j.cognition.2018.10.019 30389144 10.1016/j.cognition.2018.10.019

[CR56] Zvyagintsev M, Parisi C, Mathiak K (2017) Temporal processing deficit leads to impaired multisensory binding in schizophrenia. Cogn Neuropsychiatry 22(5):361–372. 10.1080/13546805.2017.1331160 28578638 10.1080/13546805.2017.1331160

