# Supplementary material for: Audiovisual integration of speech: evidence for increased accuracy in “talk” versus “listen” condition
Source: Exp Brain Res. 2025 May 26;243(6):154. doi: 10.1007/s00221-025-07088-7 (PMC12106506; doi:10.1007/s00221-025-07088-7)
Supplement: Supplementary file 1 — Supplementary Material 1 [file 221_2025_7088_MOESM1_ESM.docx]

**Supplementary material**

| **AV**  **onestim** | **AV**  **twostim** | **VA**  **onestim** | **VA**  **twostim** | **wholeTBW**  **onestim** | **wholeTBW**  **twostim** |
| --- | --- | --- | --- | --- | --- |
| 183 | 186 | 79 | 71 | 262 | 257 |
| 277 | 284 | 86 | 78 | 363 | 362 |
| 181 | 188 | 133 | 143 | 314 | 331 |
| 503 | 551 | 144 | 149 | 647 | 700 |
| 229 | 226 | 195 | 194 | 424 | 420 |
| 361 | 360 | 302 | 313 | 663 | 672 |
| 209 | 208 | 192 | 168 | 401 | 376 |
| 242 | 236 | 190 | 226 | 433 | 462 |

**Supplementary table 1:** Individual differences for the AV, VA and whole TBW between the passive condition with one auditory simuls and the passive condition with two auditory stimuli. Each line represents the data from each participant.

**
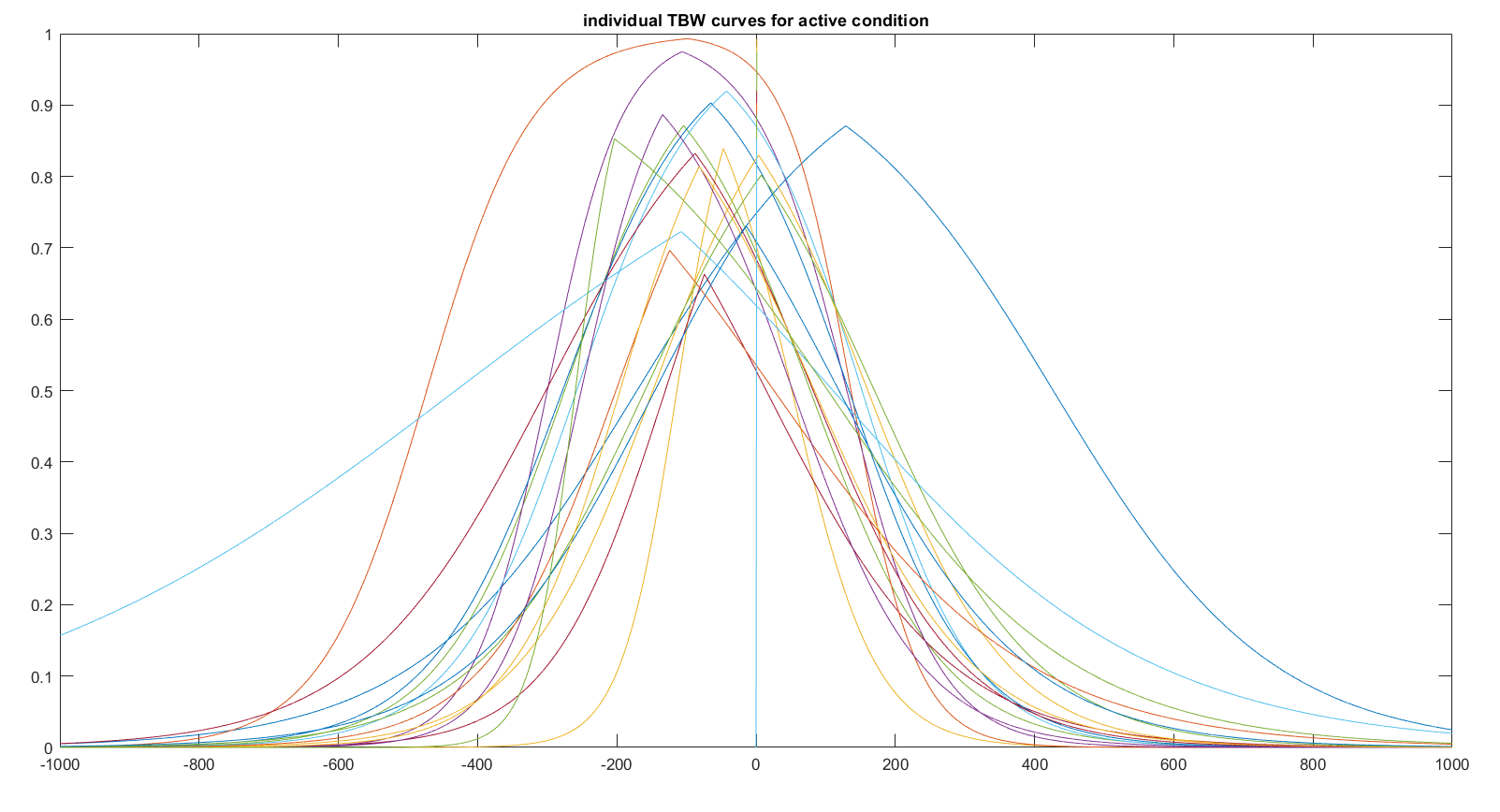
**

**
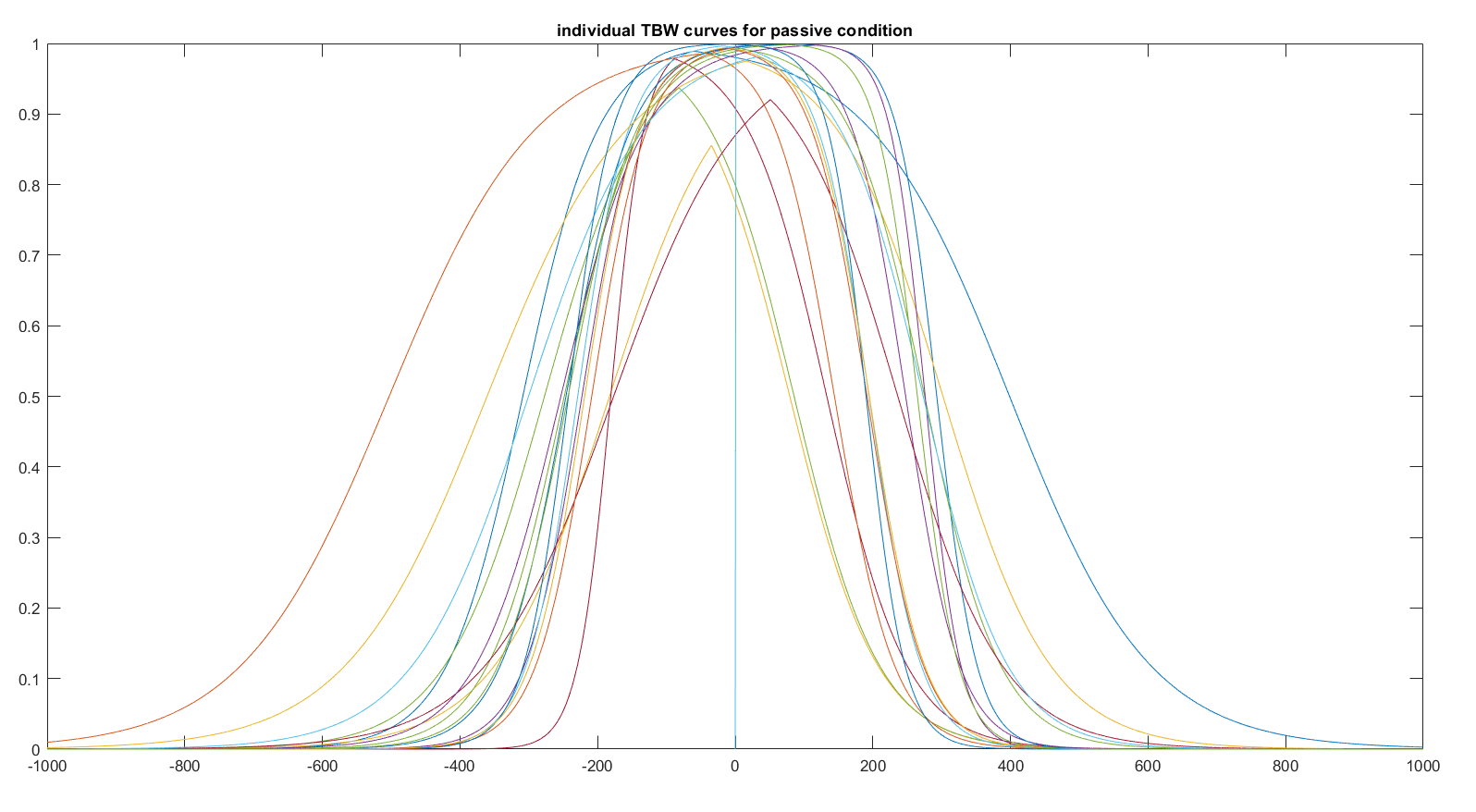
**

**Supplementary Figure 1:** Individual TBW curves for each of the 15 subjects for the active and passive condition. Each line represents the TBW of each individual. The x-axis represents the stimulus onset asynchronies (SOAs) where the negative ones indicate those where the auditory stimulus leads (AV) and the positive ones indicate those where the visual stimulus leads (VA). The y-axis represents the percentage of the synchrony reported by the participants in each SOA.
